# Epigenetics of Formaldehyde: Altered microRNAs May Be Key to Adverse Effects

**DOI:** 10.1289/ehp.119-a176b

**Published:** 2011-04

**Authors:** Cynthia Washam

**Affiliations:** **Cynthia Washam** writes for *EHP*, *Oncology Times*, and other science and medical publications from South Florida

Formaldehyde has long been associated with asthma, acute respiratory illness, and nasopharyngeal cancer. The International Agency for Research on Cancer has deemed it a known human carcinogen. A new study reveals evidence that epigenetic mechanisms may contribute to links between formaldehyde exposure and respiratory illness **[*EHP*** **119(4):494–500; Rager et al.]**. The study authors discovered that formaldehyde disrupts levels of microRNAs, or miRNAs, small regulatory molecules that play a key role in gene expression.

Ambient air contains formaldehyde given off from car exhaust, incinerators, and manufacturing and power plants. Formaldehyde also is widely used in preservatives and adhesives, including glue that binds plywood and particleboard, and it offgasses from furniture and building materials that use these products.

Despite formaldehyde’s known respiratory toxicity, little is known about its mechanism of action related to disease. The authors of this study focused on miRNAs because earlier studies linked miRNA disturbances to a number of diseases including blood and solid-tumor cancers. miRNAs act like molecular switches, turning on or off genes, including ones that lead to or protect from disease.

Nearly all the formaldehyde that humans inhale is absorbed in the respiratory tract because the gas is water-soluble and highly reactive. The authors therefore exposed human lung epithelial cells to formaldehyde gas at an air–liquid interface that mimics the human respiratory tract lining.

Of more than 500 miRNAs assessed, formaldehyde significantly downregulated 89 that are predicted to influence molecular signaling pathways relevant to cancer, inflammatory response, and endocrine system regulation, potentially representing a first step on the path toward disease. There is also preliminary evidence that the miRNAs that appeared to be most strongly affected by formaldehyde also may be altered in some cancer cells, suggesting a potential mechanism for the chemical’s carcinogenicity.

